# Immune-Mediated Inflammatory Diseases and Cancer - a dangerous liaison

**DOI:** 10.3389/fimmu.2024.1436581

**Published:** 2024-09-18

**Authors:** Jeanette A. Maier, Sara Castiglioni, Alessandra Petrelli, Rosanna Cannatelli, Francesca Ferretti, Greta Pellegrino, Piercarlo Sarzi Puttini, Paolo Fiorina, Sandro Ardizzone

**Affiliations:** ^1^ Department of Biomedical and Clinical Sciences, Università di Milano, Milano, Italy; ^2^ Department of Clinical Sciences and Community Health, University of Milan, Milano, Italy; ^3^ Gastroenterology Unit, ASST Fatebenefratelli-Sacco, Milano, Italy; ^4^ IRCCS Ospedale Galeazzi-Sant’Ambrogio, Milano, Italy

**Keywords:** Immune-Mediated Inflammatory Diseases, inflammation, immunosuppression, biologic therapy, malignancy, immune dysregulation

## Abstract

Patients with Immune-Mediated Inflammatory Diseases (IMIDs) are known to have an elevated risk of developing cancer, but the exact causative factors remain subject to ongoing debate. This narrative review aims to present the available evidence concerning the intricate relationship between these two conditions. Environmental influences and genetic predisposition lead to a dysregulated immune response resulting in chronic inflammation, which is crucial in the pathogenesis of IMIDs and oncogenic processes. Mechanisms such as the inflammatory microenvironment, aberrant intercellular communication due to abnormal cytokine levels, excessive reparative responses, and pathological angiogenesis are involved. The chronic immunosuppression resulting from IMIDs treatments further adds to the complexity of the pathogenic scenario. In conclusion, this review highlights critical gaps in the current literature, suggesting potential avenues for future research. The intricate interplay between IMIDs and cancer necessitates more investigation to deepen our understanding and improve patient management.

## 1 Introduction

The term Immune-Mediated Inflammatory Diseases (IMIDs) groups apparently unrelated multifactorial and polygenic diseases with multi-organ involvement, all sharing an aberrant, severe and continuous immune dysregulation associated with high levels of inflammatory cytokines (1). IMIDs affect a variety of organs and tissues, including the skin (e.g., psoriasis, atopic dermatitis), eyes (uveitis), joints [e.g., rheumatoid arthritis (RA)], internal lumens (e.g., inflammatory bowel disease (IBD), i.e. Crohn’s disease (CD) and ulcerative colitis (UC), asthma) (2), white and gray matter of the central nervous system [e.g., multiple sclerosis, neuromyelitis and autoimmune epilepsy (3)], and endocrine glands (e.g., type 1 diabetes (T1D), Addison’s disease). Some of these conditions arise from autoimmune mechanisms, while others result from hypersensitivity reactions. These aberrant immune responses lead to inflammation, tissue damage, and functional impairment of the involved organs or systems. IMIDs are prevalent in 5–7% of populations in developed Western countries, and, as globalization continues to expand, their occurrence is becoming more common in developing countries and among immigrant populations (4). IMIDs reduce the quality of life, are potentially disabling and represent an economic burden for health care systems. In addition, they increase the risk of developing cancer and the big question is why.

In this narrative review, we begin with a brief overview of the common etiological factors in IMIDs and cancer and then focus on the association between cancer and three common IMIDs, such as IBD, RA and T1D, and cancer. We performed a worldwide review of studies on IMIDs and cancer using three electronic medical databases, i.e. PubMed, EMBASE, and Web of Science. We selected the following keywords: “Immune-Mediated Inflammatory Diseases”, “IMIDs”, “IBD”, “RA”, “rheumatic diseases”, “T1D”, “cancer”, and “malignancy”. We included studies published in English, with available abstracts, and excluded case reports.

## 2 Common etiologic factors in IMIDs and cancer

**Figure 1:**
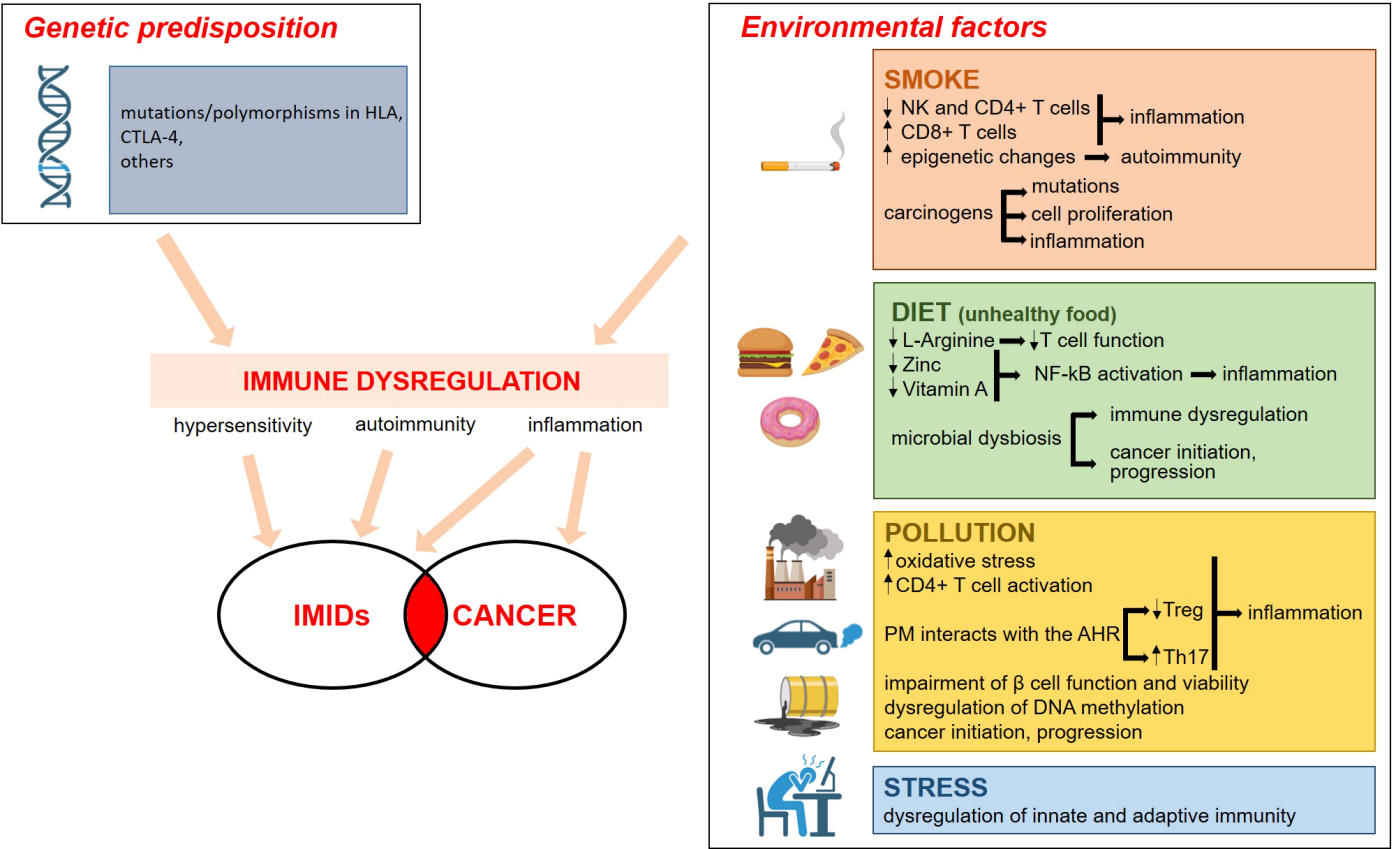
Genetic and environmental factors contribute to the etiopathogenesis of IMID. The environmental factors that promote IMIDs are the same as those that cause cancer. The boxes on the right summarize the mechanisms involved. Everything converges on a significant dysregulation of the immune system, with inflammation being a key event. For details see the text. Image created with BioRender.com.

Environmental factors play a role in the etiology of IMIDs in genetically predisposed individuals. Epidemiological studies have highlighted smoking, dietary habits, drugs, microbial dysbiosis, pollution and emotional stress (5) as common risk factors in the onset of IMIDs. Intriguingly, these environmental factors have a role also in tumorigenesis (6) (**Figure 1**).

A clear association has been reported between smoke and RA, CD, and psoriasis (6) mainly because of immune dysregulation. Indeed, active tobacco smoking impacts the immune system by decreasing circulating natural killer and CD4+ T cells and increasing CD8+ and CD8+ memory lymphocytes (7). Moreover, smoke endorses epigenetic changes to trigger the development of autoimmunity (8). The causal relation between smoke and cancer is well established, and is due to the fact that several of its components are carcinogens that cause permanent somatic mutations while others promote cell proliferation and/or prompt inflammation (9).

Processed foods, additives as well as deficiencies in micro- and macronutrients trigger inflammatory responses and disrupt immune response (10). For example, specific nutrients play key immunoregulatory roles. L-Arginine, for instance, acts as a critical nutrient and signaling molecule that shapes immune responses through the production of nitric oxide, T cell activation, immune cell proliferation, and modulation of immune suppression (11). Accordingly, L-Arginine depletion impairs anti-tumor immune responses, primarily by compromising T cell function (12). Additionally, zinc and vitamin A exhibit anti-inflammatory effects through the inhibition of the nuclear factor (NF)-kB pathway and also control the rate of antibody synthesis (13). Indeed, zinc and vitamin A depletion disrupts normal cellular processes, impairs immune surveillance, and promotes environments conducive to cancer development and progression. Nutrition also shapes the microbiota, the community of microorganisms that colonize the skin, the gut, and the respiratory mucosa (14). The microbiota influences tissue homeostasis and metabolism as well as the development of several immune cells including Th1, Th2, Th17, and immunosuppressive Treg cells (15). Not unexpectedly, gut microbiota is significantly different in IMIDs’ patients vs healthy individuals (16) and, while each specific disease has its own characteristic microbic signature, there are common dysbiotic alterations in different IMIDs (16). Considering that the microbiota also affects cell proliferation and death, it is not surprising that its composition in cancer-associated areas is different from the neighboring healthy tissue (17, 18). Accordingly, the microbiota is mechanistically involved in cancer initiation, progression, metastasis, and response to therapy (19). While some microorganisms produce genotoxins and reactive oxygen species, leading to oxidative stress and direct DNA damage (20), others release metabolites that promote tumor cell proliferation (21, 22). Moreover, dysbiosis disrupts the immune system balance by regulating immune cell activity, including T-cells and regulatory cells, thus impairing the body’s ability to fight malignancies (23). It is also relevant to highlight that some bacteria metabolize chemotherapeutic drugs, thus inducing resistance (24), and that dysbiosis impairs immunotherapy by creating an immunosuppressive environment (25). Therefore, studies should be encouraged to individuate microbial drivers in the progression from IMIDs to cancer.

Pollution is emerging as a novel player in IMIDs (26). Beyond inducing oxidative stress and long-term inflammation, pollutants dysregulate DNA methylation and Particulate Matter (PM) interacts with the Aryl hydrocarbon receptor (AHR) pathway, known to be involved in inflammatory processes and adaptive immune responses. In particular, the binding to AHR decreases Treg while augmenting Th17. Pollutants activate CD4+ T lymphocytes with the consequent increased production of pro-inflammatory cytokines, and also induce epigenetic modifications in T cells. For instance, the incidence of RA is higher in urban than in rural areas and this is due to high concentrations of PM2.5 and nitrogen dioxide (NO_2_) (27). Increased exposure to NO_2_, PM2.5 and ozone (O_3_) during childhood is also associated with increased risk of overall IBD (28). Persistent organic pollutants might play a role in T1D etiology, because they impair β cell function and viability (29).

Pollutants are also implicated in tumorigenesis. Some of them damage DNA (30), thereby activating oncogenic mutations, while others, such as PM2.5, promote cancer by inducing the expansion of cells with pre-existing oncogenic mutations (31). It is intriguing that lung malignancy is more frequent in patients with RA (32, 33), an issue that raises the question about the links between PM2.5 and the onset of the disease eventually complicated with the development of lung cancer.

Also chronic psychological stress accounts for a place in the pathogenesis of IMIDs (34–36), since it dysregulates innate and adaptive immune responses (37, 38). Moreover, the diagnosis of IMIDs engenders chronic psychological stress that, on one side, might generate mental health concerns (39), on the other one sustain chronic inflammation.

Both in IMIDs and in cancer, genetic predisposition is pivotal. Over the last twenty years, the genetic landscape of IMIDs has been intensely explored. The human leukocyte antigen (HLA) complex, located on chromosome 6, encodes proteins that play a crucial role in regulating the immune system by presenting antigens to T cells. Variations or polymorphisms in HLA genes are strongly associated with susceptibility to many IMIDs, because they influence how the immune system recognizes self versus non-self, leading to immune dysregulation and chronic inflammation in IMIDs. Moreover, hundreds of non-HLA genetic variants have been unraveled (40). For example, the locus containing T lymphocyte-associated antigen (CTLA)-4 is associated with many IMIDs (41). Accordingly, targeting the CTLA-4 pathway, often used in tumor immunotherapy, leads to multi-organ autoimmune reactions (42, 43). Genome-wide association studies have demonstrated a remarkable overlap in the loci predisposing to IMIDs (44, 45). It is known that, differently from autoantibody-negative IMIDs, such as psoriasis and CD, autoantibody-positive IMIDs, among which RA, strictly cluster with each other, as demonstrated by the evidence that, among the 150 genetic loci associated with RA, only a few are specific to the disease (46). All this knowledge has disclosed novel pathways implicated in the pathogenesis of IMIDs and has pinpointed the involvement of genes that might offer insights into the higher risk of developing cancer in IMIDs patients.

## 3 Immune dysregulation in IMIDs: an overview

Innate and adaptive immune dysregulation, driven by environmental factors in genetically predisposed individuals, is central to generate cytokine dysregulation, the decisive event in the pathophysiology of IMIDs. Cytokine signature hubs have been described in single IMIDs (2). However, an appraisal of the distinct mechanisms involved in each individual disease is beyond the scope of this article. Our aim is to provide a synthetic overview on the common aspects of the complex immune dysregulation occurring in IMIDs and the connections with cancer.

In addition to the classical pro-inflammatory cytokines interleukin (IL)-1, IL-6 and Tumor Necrosis Factor (TNF)α, whose role in IMIDs has been amply described (47), the IL-23/17 axis is emerging as a common feature in several IMIDs among which IBD, psoriasis, uveitis, psoriatic arthritis and evidence are accumulating about its role in RA (48, 49). Upon exposure to a pro-inflammatory milieu, IL-23 is synthesized by several types of cells, including dendritic cells and macrophages, and acts on IL-23 responsive cells, which include neutrophils, natural killer lymphocytes, mast cells, macrophages, memory T cells, all localized at the barrier surface, and also cells involved in transmitting biomechanical forces (50–52). IL-23 promotes the release of IL-17, which induces pro-inflammatory mediators and cooperates with other molecules in triggering and chronicizing inflammation (53). By transcriptional and post-transcriptional regulation, IL-17 stimulates the release of TNFα, a downstream effector common to many IMIDs (2), IL-1, IL-6, IL-8 and other cytokines and chemokines, thus unbalancing the complex communication network that, tightly tuned (54) in physiological conditions, is radically deregulated in IMIDs. As an example, IL-1 acts synergistically with IL-23 to perpetuate the continuous high production of IL-17 (55) and, consequently, chronic inflammation (55). Therefore, one would expect the inhibition of IL-17 to be a success, but this is true in psoriasis and ankylosing spondylitis, and not in CD (56, 57) where, paradoxically, the clinical course is aggravated. Similarly, in spite of the fact that TNFα is upregulated in most IMIDs, not all the patients respond to anti-TNFα therapy, and many of the initially responders lose response over time (58). It is likely that anti-TNFα antibodies promote a change in innate and immune cell infiltrates so that TNFα independent inflammatory pathways emerge and keep the disease active.

It is worth mentioning that chemokines are important actors in several IMIDs, from RA (59) to IBD (60), from LSE (61) to psoriasis (62) and T1D (63), as they recruit immune cells into the tissues and regulate their reciprocal interactions. Of note, the chemokine/chemokine receptor axis is also implicated in tumorigenesis, because it controls cell proliferation, stemness, survival and neovascularization, and contributes to the generation of an immunosuppressive tumor microenvironment (64).

Dysfunction of immune checkpoints in IMIDs is beginning to draw some attention. An imbalance between co-stimulators, such as CD28 and CD40, and co-inhibitors, among which CTLA-4 and programmed cell death (PD)-1, contributes to immune deregulation and inflammation (65), as demonstrated by immune-related adverse effects, which include IBD and dermatitis (66), experienced by individuals receiving checkpoint inhibitors to treat malignancies (67). CTLA-4, expressed by activated and regulatory T lymphocytes, has a relevant role in maintaining immune homeostasis. Abatacept, a fusion protein consisting of the extracellular domain of CTLA-4 and a genetically engineered fragment of the Fc region of human immunoglobulin G1 (IgG1), is efficacious in RA, because it inhibits the co-stimulation of T cells (68). In T1D it modifies the pattern of immune cells and enhances insulin secretion, but it does not delay the progression to glucose intolerance (69). In IBD (70) and psoriasis (71), abatacept is not effective, thus highlighting on one side the complexity of approaching IMIDs, on the other the current gaps in our knowledge. PD-1 is another immune checkpoint receptor which is expressed predominantly by T lymphocytes (72). When PD-1 interacts with its ligands PD-L1 or PD-L2, it elicits an inhibitory response by targeting T cell receptor signaling (73). Since many malignant cells overexpress the PD-1 and its ligands, this pathway is a target for immunotherapy. PD-1 is upregulated in peripheral T lymphocytes in RA (74) and in the professional immune cells of the lamina propria in IBD (75). However, a reduced binding of PD-1 by PD-L1 may down-regulate pathogenic immune responses. Indeed, a phase 2 trial in patients with RA has recently shown that the PD-1 agonist monoclonal antibody peresolimab is safe and improves the clinical course of the disease (76).

## 4 Chronic inflammation in IMIDs and cancer

The connection between IMIDs and some cancers is well known, but the mechanisms involved remain unclear and are often controversial. As mentioned above, infection, diet, environment are common to IMIDs and cancer, and lead to chronic inflammation that plays the lion’s share in the development of malignancies.

A link between inflammation and cancer has been appreciated for a long time, since when Rudolf Virchow wrote that “chronic irritation and inflammatory hyperplasia predispose to cancer development” (77). Nowadays, prolonged inflammation is considered one of the hallmarks of cancer (78). An inflammatory microenvironment can contribute to tumorigenesis by increasing oxidative stress, which damages DNA, by activating prosurvival pathways and promoting growth, migration, invasion of tumor cells, and also angiogenesis, thereby supporting tumor progression locally and at metastatic sites (79–81).

The altered intercellular communication due to the upheaval of the cytokine network has a prominent role in the progression toward neoplasia. Inflammatory cytokines activate the transcription factor NF-kB, which on one side fuels cytokine production, on the other inhibits epithelial apoptosis. They also induce another transcription factor, i.e. STAT3, which not only contributes to the maintenance of an inflammatory environment but also acts on the epithelium stimulating growth and protecting from apoptosis (82). In addition to the prototypical inflammatory cytokines such as TNFα, IL-1s and IL-6 whose role in cancer has been largely described, novel players are entering the scene. The IL-23/17 pathway, which is implicated in several IMIDs, not only promotes and maintains inflammation, but also weakens the barrier function of the skin, gut and lung, and reduces CD8+ lymphocyte antitumor immunosurveillance, both factors that contribute to cancerogenesis (53). A seminal finding is that IL-17A is necessary and sufficient to activate the hypoxia inducible factor (HIF)1α (83), thus demonstrating the coupling of inflammatory, metabolic, and migratory programs as well as angiogenesis (84), all events clearly involved in cancer. IL-17 also stimulates epithelial stem cell proliferation after injuring the tissue with a carcinogenic agent (85) and the inhibition of IL-17 prevents colon cancer in an experimental murine model of colitis (86).

Attention has been devoted also to IL-36, a member of the IL-1 superfamily, which is upregulated in the synovium of patients with RA, in psoriatic skin, in the mucosa of patients with IBD, in the sera of patients with SLE (87–89). Its role in cancer is controversial, as it displays both anti and pro tumor properties depending on the type of neoplasm and its level of expression (90). IL-36 markedly increases and exerts pro-tumorigenic effects in lung and colorectal cancers (91, 92). Of interest, colon cancer cells without the IL-36 receptor grow slower and express lower amounts of Ki-67 than controls (91). A recent study shows that increased IL-36 expression is associated with a decrease of 5 year survival rates in colon cancer patients (92).

## 5 Common cues in IMIDs and cancer

While in the early stages of cancer the immune system identifies and controls the tumor cells, in the later stages anti-tumor immune cells are corrupted into tumor-promoting immune cells that sustain survival, growth, invasiveness of tumor cells and generate chronic inflammation. In the end, tumor cells escape immune surveillance through the activation of various anti-detection pathways (93). These events are orchestrated by the cytokine storm generated within the tumor microenvironment (94–96). Among the various mechanisms of evasion of anticancer immunity, a light is shed on myeloid-derived suppressor cells (MDSC), which derive from neutrophils and monocytes in response to high levels of inflammatory cytokines (66, 97). MDSC are potent inhibitors of immune responses mediated by natural killer cells, B and T lymphocytes, thus facilitating the escape of tumor cells. The number of MDSC rises in various pathological conditions, such as cancer, inflammation, and transplantation (98, 99). Notably, MDSC accumulate in the lesions occurring in IMIDs and their number is proportional to the severity of the disease (100). The number and activity of MDSC are increased in the blood of T1D, RA and IBD patients (101–103). To the best of our knowledge, there are no studies correlating MDCS, IMIDs and cancer risk, albeit this issue is very challenging and deserves further investigations. The link between autoimmunity and neoplasia is further supported by increased risk of lymphoma and gastric cancer in individuals with mutated CTLA-4 and, consequently, with dysregulated immune responses (104).

It should also be underscored that tissue damage in IMIDs triggers a reparative response that represents a double edged sword. Whereas the primary aim is to heal the injured tissue, an exuberant and unleashed production of growth factors together with the myriad of inflammatory mediators overstimulates cell proliferation, bolsters transformation, and promotes the development of dysplasia that can progress into malignancy (105). Also stromal cells play a role in the attempt of healing the tissue and, eventually, in the onset of cancer. Fibroblasts and myofibroblasts deposit collagen, fibronectin, laminin and continuously remodel the extracellular matrix by releasing proteases in IMIDs and, even more, in cancer (106).

Another common event in IMIDs and cancer is pathological angiogenesis. Inflammation and cancer share molecules that support the formation of a new vascular network, such as prostaglandins, cytokines, chemokines and growth factors, with a prominent role of the members of the vascular endothelial growth factor (VEGF) family (107), secreted by platelets, activated T lymphocytes, neutrophils, macrophages, dendritic cells and tumor cells.

The inflammatory environment suffices to explain the strong association existing between organ-specific immune-mediated diseases and the risk of local cancers, in agreement with the idea of tumors as wound that do not heal (108). However, IMIDs moderately increase oncologic risk also in distant organs and in different systems (see below). Again, lifelong immune dysregulation and altered cytokine profile are likely to be implicated.

Another interesting, albeit overlooked, issue is that cytokines also activate the hypothalamic–pituitary–adrenal axis (109), significantly shaping immune function and consequently inhibiting antitumor immune responses. This effect is further magnified by the common anxiety or depression experienced by patients with IMIDs, who must live with a chronic, recurrent, and disabling disease (110).

## 6 IMIDs and cancer: the example of IBD

It is well known that IBD predispose to intestinal cancers, a finding that does not surprise because the persistent activation of the transcription factors NF-kB and STAT3 in the lesions fuels inflammation and upregulates genes implicated in tumor cell survival, proliferation and invasion. Intestinal inflammation can also affect the brain through the brain-gut axis, resulting in the activation the hypothalamic–pituitary–adrenal system, which impairs the antitumor immune defenses and promotes cancer occurrence. Indeed, malignancies are the second most common cause of death in IBD patients after cardiovascular diseases both in male and female (111).

**Figure 2:**
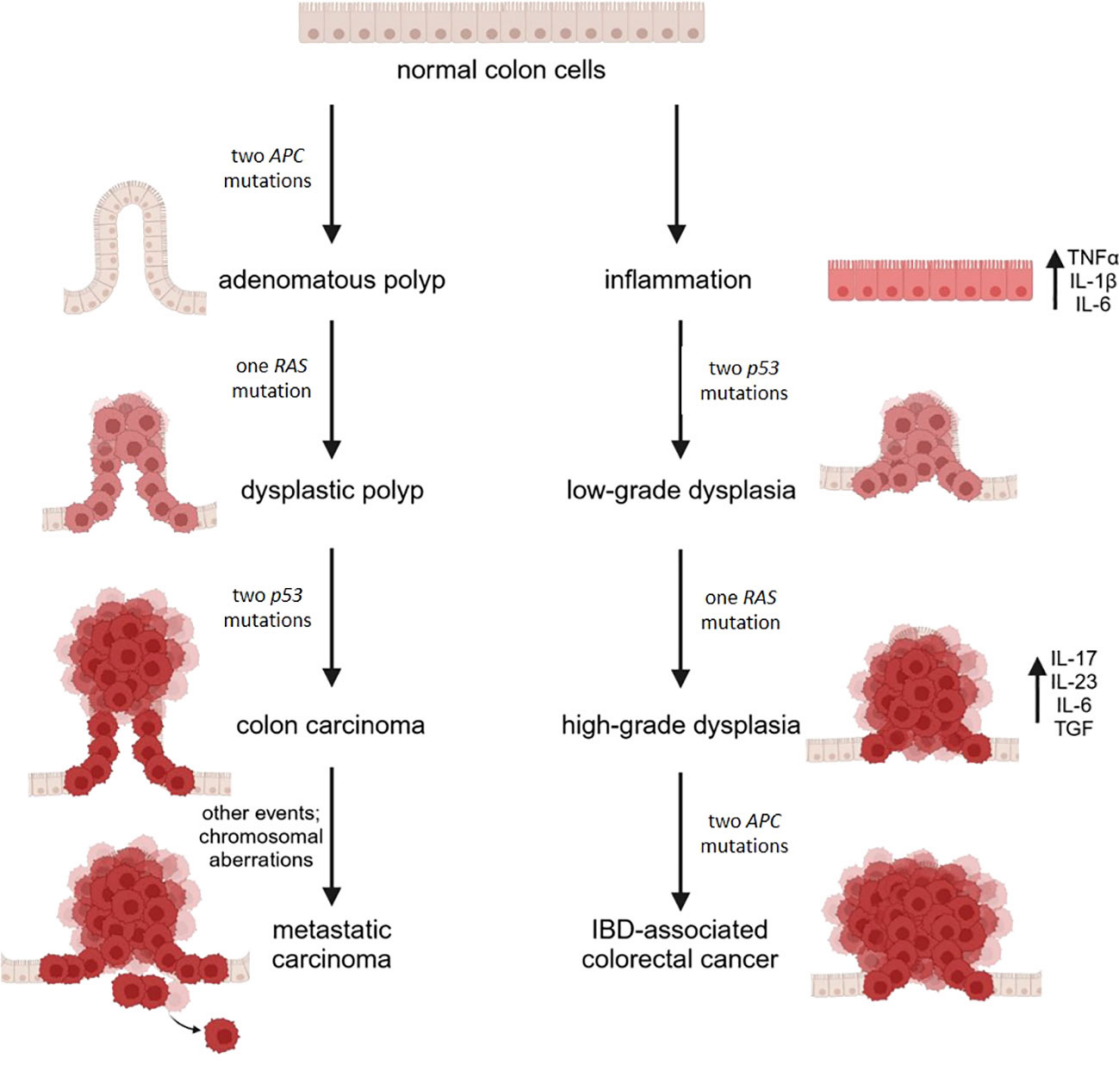
A schematic diagram illustrating the molecular mechanisms in sporadic (CRC) and IBD-related colorectal cancer. CRC arises from the accumulation of mutations in oncogenes and tumor suppressor genes, driving the progression from single preneoplastic cells to adenoma and ultimately to carcinoma. In contrast, IBD-related colorectal cancer is driven by chronic inflammation, which leads to the production of proinflammatory cytokines and the buildup of reactive oxygen and nitrogen species, promoting genomic aberrations and instability. This process results in mutations in oncogenes and tumor suppressor genes. Mutations in p53 lead to low-grade dysplastic mucosa, with subsequent mutations in KRAS contributing to the progression from low-grade to high-grade dysplasia. Finally, mutations in the APC gene culminate in the development of cancer. Image created with BioRender.com.

Long-standing UC and CD colitis cause an approximately 2–3-fold increased risk of colorectal cancer (112). Typically, neoplasms develop from dysplasia originating on inflamed areas, in the sequence inflammation-dysplasia-adenocarcinoma differently from the adenoma-carcinoma sequence described in sporadic colon cancer (**Figure 2**). Accordingly, dysplasia is the most reliable marker of increased risk of colon cancer in IBD (113). Of note, mutant cells bearing genomic and epigenomic alterations are detectable even before the onset of dysplasia. Apart from the low rate of KRAS mutations, in IBD associated colon cancer driver genes are the same as in sporadic colon cancer, but the timing of the mutations is different. P53 mutation or silencing occurs very early in the process, eventually before the onset of dysplasia, whereas APC is mutated or lost later and less frequently than in sporadic colon cancer (114). It is noteworthy that genome wide studies demonstrate an important increase in mutations in the 5’ untranslated region of p53 in IBD associated colon cancer (115). The same study individuates as a unique feature in IBD colon cancer the hypermethylation and consequent loss of function of the polymeric immunoglobulin receptor (PIGR), which is responsible for the transport of IgA and IgM through the epithelium (116). This event can be interpreted as a loss of the epithelial properties of tumor cell, as further supported by the downregulation of genes promoting epithelial differentiation. In parallel, genes involved in modeling the extracellular matrix are upregulated. All together these alterations facilitate the acquisition of a mesenchymal colon cancer subtype. This subtype is linked to drug resistance (117), reduced survival and is characterized by the presence of a high number of Treg, indicating that the microenvironment is highly immunosuppressive (118). In the case of colon cancer arising in patients with UC, a systems biology approach (119) revealed the upregulation of two chemokines, namely CXCL1 and IL-8, the matrix metalloproteinase (MMP)-7, the serine protein urokinase-type plasminogen activator, the tissue inhibitor of metalloproteinase (TIMP)-1, and the solute carrier 16 member 9 (SLC16A9) which transports monocarboxylic acids. This molecular signature is proposed as specific for colon cancer in UC and further corroborates the dominant role of inflammation and matrix remodeling pathways.

CD is also associated with a high risk of developing small bowel adenocarcinoma (120), which occurs mainly in young adults and has a poor prognosis (121). KRAS mutation and p53 loss of function are common characteristics of sporadic and CD related small bowel cancer, whereas p16 positivity, the nuclear accumulation of β-catenin and mutations of isocitrate dehydrogenase (IDH)-1 seem to be specific in CD small bowel cancer (122).

Although much rarer, intestinal B cell lymphoma, which is uncommon in the general population, is a dreadful complication in IBD patients, mainly in male older than 65, after a mean average of 12 years from the diagnosis (123). Chronic inflammation, therapy with the immunosuppressant thiopurine and Epstein Barr virus positive lymphocytes are the main driving factors for uncontrolled B cell proliferation ultimately progressing to lymphoma.

Patients with long lasting IBD have a slightly increased risk of extralocal malignancies (124), among which cholangiocarcinoma (125, 126). It frequently develops in the context of primary sclerosing cholangitis, a chronic inflammatory disease, according to the inflammation-dysplasia-cancer sequence. Even though the pathogenic mechanisms remain unclear, it is reasonable to propose the involvement of altered microbiota and metabolism in the inflamed gut, along with an altered bile acid profile due to impairment of the enterohepatic axis, as described in IBD (125). Moreover, a recent metanalysis of cohort studies confirms previous reports about the increased risk of prostate cancer in IBD, in particular in European UC patients (127). In addition to the tumor promoting role of the aberrant microbiome in UC, chronic intestinal inflammation fosters genetic instability and the upregulation of pro-cancer signaling pathways in the prostate (128).

## 7 IMIDs and cancer: the example of rheumatic and musculoskeletal diseases

The association between rheumatic and musculoskeletal diseases (RMDs) and neoplasm is a dynamic and continuously evolving field of scientific research. While certain RMDs, such as RA, SLE, Systemic Sclerosis (SSc), Sjögren’s syndrome, and inflammatory myopathies are recognized to have a heightened association with an increased risk of cancer (129), the underlying mechanisms remain multifaceted. Undoubtedly, a significant factor contributing to the increased cancer risk is the presence of chronic inflammation and associated tissue damage (130). For instance, the risk of lymphoma in patients with Sjogren’s syndrome is known to correlate with disease activity and severity (131). Similarly, in the case of RA, elevated markers of inflammation, such as erythrocyte sedimentation rate and C-reactive protein, have been associated with an increased risk of neoplastic disease (129, 132).

Another hypothesis suggests that natural immune responses against cancer cells may trigger autoimmunity and rheumatic disease (133). This theory is supported by cases where cancer rapidly develops in patients with dermatomyositis and SSc, often occurring within three years of the onset of autoimmune disease. Moreover, the presence of specific autoantibodies, such as anti-RNA polymerase III and anti-RNA-binding region-containing protein 3 (RNPC3) in SSc (134, 135), or anti- transcription intermediary factor 1 (TIF1)-γ and anti-nuclear matrix protein 2 (NXP2) in dermatomyositis, could help to stratify patients at higher risk of cancer, as a strong association has been demonstrated (136, 137). In addition to inflammation and immune response, the inability to clear viral infection, as observed in SLE, can also elevate the risk of certain cancer. For example, the higher susceptibility of SLE patients to human papillomavirus infection is believed to contribute the increased risk of cervical cancer in this population (138).

Sjögren’s syndrome is primarily associated with an elevated risk of developing lymphoma, notably non-Hodgkin lymphoma (with a prevalence of around 5%). The risk of lymphoma is estimated to be 5-10 times greater than that of the general population (139). However, a recent meta-analysis by Zongh et al. showed that patients with Sjögren’s syndrome also have an increased risk of solid tumors, such as lung, thyroid, and non-melanoma skin cancers (140).

Individuals diagnosed with inflammatory myopathies have long been recognized as having a higher likelihood of developing specific cancer types, with adenocarcinoma being the predominant histological tumor type (141, 142). The period of greatest cancer susceptibility occurs within three to five years before and after the diagnosis of myositis, and cancer risk appears to be contingent on the specific subtype of inflammatory myopathy. Individuals with dermatomyositis exhibit a 5.5-fold increased cancer risk, while those with polymyositis display a 1.6-fold elevation (142–144).

SSc is characterized by an increased age- and sex-adjusted risk of developing cancer, often ranging from 1.5 to 4 times higher than that of the general population (145, 146). The relationship between SSc and cancer risk is thought to be related to the damage caused by SSc in various body sites, potentially predisposing individuals to malignant transformation. This may explain why esophageal and lung cancers are more frequently observed in these patients, given the association of gastroesophageal reflux and interstitial lung disease with the pathology (147, 148).

There is evidence suggesting association between SLE and an increased susceptibility to certain malignancies (149, 150). Epidemiological studies have indicated that individuals with SLE face a moderately elevated risk of cancer, particularly hematological malignancies such as non-Hodgkin lymphoma (150). Additionally, a higher prevalence of cervical dysplasia and cervical cancer has been observed in women with SLE (151).

A recent meta-analysis indicated that individuals with RA may have a slightly increased risk of cancer (152). In particular, lymphoma and lung cancer are the most commonly observed types of neoplasm in this group of patients (153). This elevated risk is believed to be influenced by shared risk factors. such as smoking, in addition to the mechanism of chronic, persistent inflammation (154, 155).

In conclusion, the intricate relationship between neoplastic diseases and RMDs is an ongoing subject of scientific investigation. This complex connection highlights the need for more comprehensive data and further research to elucidate the underlying biological mechanisms. A deeper understanding of these mechanisms is essential for improving the management and care of patients with both RMDs and cancer.

## 8 IMIDs and cancer: the example of type 1 diabetes

Type 1 diabetes (T1D) is a chronic autoimmune condition characterized by the destruction of insulin-producing β cells in the pancreas (156). Emerging evidence suggests a link between T1D and cancer risk, but the relationship is multifaceted and not fully understood. Numerous studies have investigated the standardized mortality ratio for cancers among patients with T1D compared to the general population. These reports have yielded conflicting results (157–159), often due to limitations in statistical power, which affect the precision of risk estimates for specific cancer types.

In 2016, Carstensen et al. conducted an extensive study analyzing cancer incidence in individuals with T1D using population-based registries in five countries (160). Their findings revealed that hazard ratios (HRs) for all cancers combined were slightly elevated in both men (HR 1.01) and women (HR 1.07) with T1D. Notably, elevated HRs were observed for cancers of the liver, pancreas, and kidney. Conversely, prostate cancer (HR 0.56) and breast cancer (HR 0.90) exhibited reduced risks in men and women with T1D, respectively. Interestingly, the risk of some cancers in individuals with T1D appears to resemble that in people with type 2 diabetes. Factors such as high blood sugar levels may contribute to the elevated cancer risk in both types of diabetes. Additionally, emerging contributors, such as obesity and insulin resistance - conditions for which growing evidence indicates increased incidence in symptomatic and pre-symptomatic T1D individuals (161–163) - may also play a significant role in cancer development.

An intriguing explanation of the link between T1D and cancers lies in the relationship between daily insulin dose and cancer risk. A recent study revealed that higher daily insulin doses are associated with an increased risk of cancer, even after adjusting for age and sex (164). Both *in vitro* and *in vivo* studies have highlighted the pivotal role of insulin and the insulin receptor in cancer biology (165). Hyperinsulinemic states contribute to increased hepatic insulin-like growth factor (IGF)-1 production through the upregulation of the growth hormone receptor (GHR) and enhanced GHR signaling (166), demonstrating the potential to induce cancer cell proliferation and their capacity to spread to secondary sites (167). However, epidemiological data on the link between disease duration and cancer in T1D are inconsistent, with some studies indicating that cancer risk is highest at the time of diabetes diagnosis and decreases over time (160), while others report cancer development in patients with a mean diabetes duration of 25 years (164).

Hyperglycemia promotes tumorigenesis through the “Warburg effect,” which involves increased glucose uptake by cancer cells to fuel their proliferation (168, 169). This can contribute to the cancer predisposition associated with diabetes (170). Furthermore, hyperglycemia stimulates the production of advanced glycation end products (AGEs), which interact with their receptor, RAGE, to activate NF-kB and generate reactive oxygen species (171). This cascade accelerates oxidative stress, leading to increased proinflammatory signaling and potentially promoting transformation (172, 173).

In conclusion, the relationship between T1D and cancer risk is intricate and influenced by various factors, including insulin dose, disease duration, and the complex interplay of metabolic pathways. As research continues, a deeper understanding of these mechanisms may shed light on strategies for cancer prevention and improved care for individuals with T1D.

## 9 IMIDs therapeutics: a role in cancer?

The relationship between immunosuppressive drugs and risk of malignancies has been widely explored, but the results are still conflicting (174).

Historically, immunomodulators such as thiopurines and methotrexate were the milestone of treatment of IMIDs. In this setting, the longstanding experience in transplanted patients showed an increased risk of skin cancer such non-melanoma skin cancers (NMSC), and lymphoproliferative diseases associated to Epstein-Barr virus infection (175). In the past, the large use of azathioprine and 6-mercaptopurine in IBD patients confirmed this association (176–178), and in 2009 the large prospective study by Beaugerie et al. (176) found that IBD patients receiving thiopurines showed an hazard ratio (HR) of 5.28 (2.01-13.9, p=0.0007) of developing lymphoproliferative disorders compared to other IBD patients.

Data on methotrexate are controversial: a systematic review in 2010 found an increased risk of melanoma in RA and NMSC in patients with psoriasis (179), but other studies did not confirm it (180, 181). Recent evidence suggests a higher rate of NMSC associated with the use of methotrexate, demonstrating a dose-response pattern (182). Similarly, cyclosporine can also increase the risk of skin cancer in patients with psoriasis (183). Studies in IBD patients are not available; however, effects are likely to be the same.

On one hand, these drugs may promote the development of malignancies through direct DNA modifications and by altering immunosurveillance of tumor cells or mutagenic viruses (184–186). On the other hand, controlling inflammation with these drugs is one of the primary strategies for cancer prevention in some gastrointestinal malignancies among IBD patients (187).

In the late 1990s, the introduction of biologic therapies revolutionized treatment approaches. Initially, their ‘targeted’ effects were considered to ensure safety, and short-term cancer risks were thought to be minimal. However, long-term effects were unpredictable at that time and remain controversial today. Early studies on the association between anti-TNFα therapies and malignancies reported a possible link to higher rates of lymphoma and melanoma (188, 189). However, more recent studies on anti-TNFα therapies appear to exclude a link with increased risk of cancer in RA (190), psoriasis (191), and IBD (192).

According to the phase 2/3 studies, the use of selective agents such as the anti-integrin vedolizumab and the anti IL-12/23 ustekinumab does not carry any risk of cancer development (193, 194) and they are considered safe in patients with a prior history of cancer (195, 196). Regarding antibodies targeting IL-23, risankizumab modestly increased oncologic events, with a clear prevalence in men (197), while cancer occurred in a small number of patients treated with mirikizumab (198). Due to the quite recent introduction of these drugs, long-term effects are still unknown.

Recently, small molecules such the Janus kinase (JAK) inhibitor tofacitinib were approved as therapeutic option in IMIDs patients (199). Clinical trials showed a higher incidence of malignancies compared to anti-TNFα or general population, especially in RA, not confirmed in real life studies and in clinical practice (200, 201). An increased short term risk for NMSC was described in patients with RA or psoriatic arthritis initiating treatment with the JAK inhibitors tofacitinib, baricitinib (202), filgotinib (203) and upadacitinib (204). This latter molecule increases the risk of NMSC in a dose dependent manner (204).

Thus, as available data are still limited and controversial, more long-term studies are needed to confirm this association.

## 10 Gaps and future directions in research on IMIDs and cancer

In spite of the significant advances in our understanding of the link between IMIDs and cancer, there is still a long way to go for researchers and clinicians.

Studies should be fostered to highlight whether sex or ethnicity impact the progression from IMIDs to local and distant malignancies. It is also important to identify molecular markers involved in this progression, as recently demonstrated by a system biology approach in UC patients [108]. These markers can function as prognostic tools and therapeutic targets. It is clear that intercellular communication goes awry in IMIDs and until now attention has been devoted mainly to soluble molecules and far less to exosomes. These 30-150 nm sized vesicles are released by many cells and contain lipids, proteins and nucleic acids (including non coding RNAs) that can be delivered locally as well as to distant districts. They regulate immune system, remodel the extracellular matrix and other crucial biological processes (205). Exosomes can also promote chronic inflammation, facilitate immune evasion, and contribute to tumor progression (206). While their role in cancer is well established, very little is known about potential alterations of exosome characteristics in IMIDs. Therefore, it would be relevant to individuate differences in exosomes between IMIDs patients and the general population, and to investigate whether their cargo changes when malignancies arise.

Interesting insights may emerge from studies on organoids derived from biopsies or induced pluripotent stem cells of IMIDs patients. Organoids are three-dimensional, cell-based *in vitro* models that replicate the complex structure and function of tissues (207). They are useful to answer fundamental questions about disease modeling, gene expression, drug response (208) with the final aim of personalizing medical approaches. Organoids have been successfully developed from pluripotent stem cells of UC patients and shown to recap colitic reactivity (209), thus underscoring that this approach may advance diagnostics and therapy at the individual level. The question is: can organoids be helpful to predict the potential progression in neoplasia?
